# Structure–Activity Relationship of the Tyrosinase Inhibitors Kuwanon G, Mulberrofuran G, and Albanol B from *Morus* Species: A Kinetics and Molecular Docking Study

**DOI:** 10.3390/molecules23061413

**Published:** 2018-06-11

**Authors:** Prashamsa Koirala, Su Hui Seong, Yajuan Zhou, Srijan Shrestha, Hyun Ah Jung, Jae Sue Choi

**Affiliations:** 1 Department of Food and Life Science, Pukyong National University, Busan 48513, Korea; prashamsakoirala20@gmail.com (P.K.); seongsuhui@naver.com (S.H.S.); zhouyajuan26@gmail.com (Y.Z.); srijanstha003@gmail.com (S.S.); 2 Department of Food Science and Human Nutrition, Chonbuk National University, Jeonju 54896, Korea

**Keywords:** *Morus* species, mulberrofuran G, kuwanon G, albanol B, mushroom tyrosinase, structure-activity relationship

## Abstract

Kuwanon G (KG) and benzofuran flavonoids such as mulberrofuran G (MG) and albanol B (AB) isolated from *Morus* sp. are reported to exhibit anti-Alzheimer’s disease, anti-inflammatory, fungicidal, anti-cancer, anti-bacterial, and anti-tyrosinase properties. We investigated the inhibition of mono- and diphenolase activity of mushroom tyrosinase by KG, MG, and AB. KG and MG displayed acceptable inhibition activity compared to kojic acid. AB did not show any activity up to 350 µM. MG displayed six-fold higher inhibition of *l*-tyrosine oxidation (IC_50_ = 6.35 ± 0.45 µM) compared to kojic acid (IC_50_ = 36.0 µM). Kinetic studies revealed that KG and MG inhibited monophenolase activity of tyrosinase in a competitive manner. Docking simulations of KG and MG demonstrated favorable binding energies with amino acid residues of the active sites of tyrosinase. Our investigation of the structure-activity relationship of the fused benzofuran flavonoids (MG vs. AB) implicated the methyl cyclohexene ring moiety in tyrosinase inhibition. The enzyme substrate and relative structural analyses demonstrated that KG and MG from *Morus* sp. could be useful natural tyrosinase inhibitors in foods or cosmetics.

## 1. Introduction

Tyrosinase is a ubiquitous enzyme in micro-organisms, animals, and plants. The oxidation of *l*-tyrosine and *l*-3,4-dihydroxyphenylalanine (*l*-DOPA) are rate-limiting steps in the melanin formation pathway mediated by tyrosinase. These steps are crucial for skin protection against UV damage. Functional deficiencies of this pathway can result in serious dermatological diseases. Mushroom tyrosinase is a commercially available tyrosinase that has high homology with mammalian tyrosinase. Tyrosinase inhibitors are vital in the food industry [1].

Mulberry species, genus *Morus* L. of the family Moraceae, are widely distributed. Fourteen species have been reported and classified by Zeng et al. [2] and are widespread in the sub-tropical regions of Asia such as Japan, India, China, and Korea. The leaves are eaten by silkworms (*Bombyx mori* L.), are used in Chinese herbal tea [3], and are considered potent due to the presence of steroids, terpenoids, saponins, alkaloids, flavonoids, and tannins [4]. The ripe fruit is edible and used in pies, tarts, wines, cordials, and herbal teas. The leaves are sold in various forms as nutritional supplements. The mature plant contains significant amounts of resveratrol, particularly in the stem bark [5]. The leaf, root bark, and fruit of the mulberry plant have an extensive history in traditional Chinese medicine. Various food products containing mulberry leaves, such as mulberry tea, are used in many countries [6]. Mulberry has a long history as a conventional medicinal herb due to its chemical composition and pharmacological functions. Anti-diabetic [7], cardioprotective [6], antifungal [8], antioxidant [9], hepatoprotective [10], and cytotoxic activities [11] have been reported from *Morus* species.

The tyrosinase inhibitory activity of kuwanon G (KG) is unclear [12,13], but it has displayed antioxidant [14], antibacterial [15], cosmetic [13], anti-Alzheimer’s disease [16], anti-inflammatory [17,18], and anti-asthmatic [19] properties. Mulberrofuran G (MG) from *Morus* exhibited antibacterial [20], antioxidant [21], and hepatoprotective [22] activities, cosmetic value, and tyrosinase inhibition activity [12]. Albanol B (AB) has also demonstrated anti-Alzheimer’s disease [16], antibacterial [23], and antioxidant [5] activities. Tyrosinase inhibition studies have been conducted in *Morus australis* [24] and *Morus nigra* [12]. *Morus australis* was previously investigated as an anti-obesity [25] and skin whitening [26] agent. Oxyresveratrol was the prime component [24] along with anthocyanins [25], phenolic compounds [27], and flavonoids [28]. *Morus nigra* contains phenolic compounds, including oxyresveratrol and mulberroside A [12], with neuroprotective [29], antioxidant, antibacterial, and cytotoxic activities [30].

Structure–activity relationship (SAR) studies can assist in identifying active moieties for the development of novel drugs. For this, it is necessary to understand the reaction mechanism. Chao et al. [31] demonstrated the effects of essential oils comprising a methyl cyclohexene ring on melanin content and cellular tyrosinase activity, which supported our investigation of this particular moiety.

Our study mechanistically investigated the reason behind the conflicting tyrosinase inhibitory activity of KG through monophenolase and diphenolase inhibitory assays with *l*-tyrosine and *l*-DOPA as a substrate, respectively [12]. In addition, SAR analysis as well as kinetics and molecular docking studies were used to characterize the interaction of KG, MG, and AG from *Morus* species with tyrosinase for the first time.

## 2. Results

### 2.1. Inhibitory Activities of KG, MG, AB and 1-Methyl-1-Cyclohexene on Mushroom Tyrosinase (l-Tyrosine and l-DOPA Substrates)

**Figure 1:**
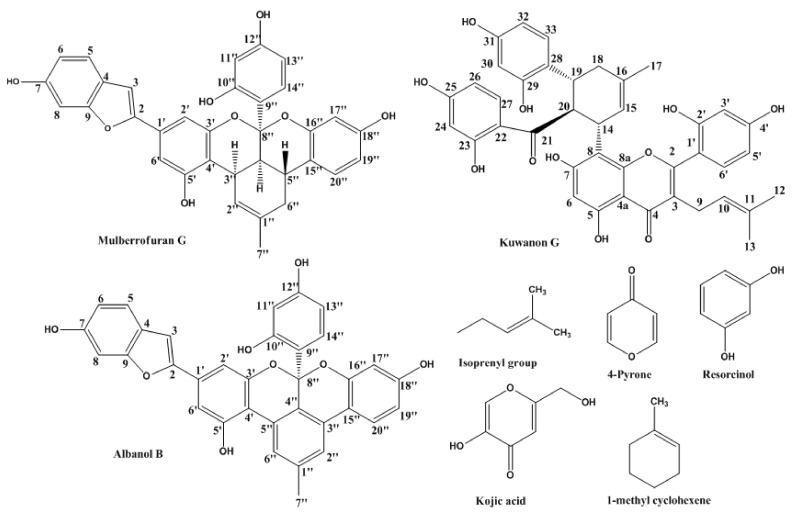
Structure of compounds from *Morus* species and structural moieties explaining structure-activity relationship.

**Figure 2:**
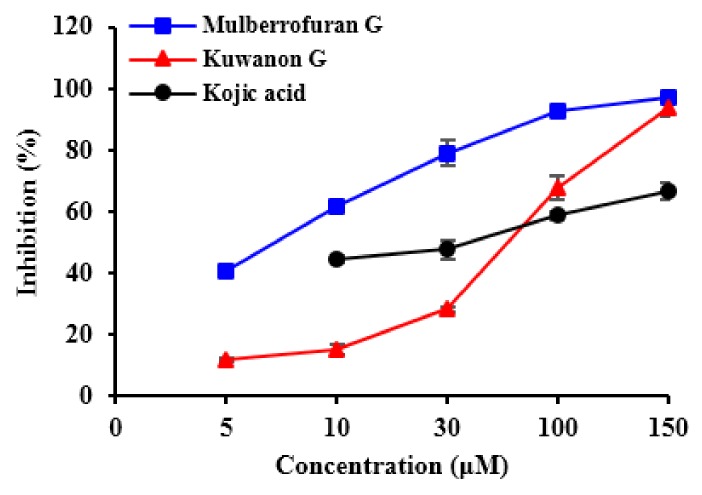
Concentration-dependent inhibition of kuwanon G, mulberrofuran G, and kojic acid on the activity of tyrosinase for the catalysis of *l*-tyrosine at 25 °C.

Three compounds from Morus species (Figure 1) were tested for their tyrosinase inhibitory activity with *l*-tyrosine (Table 1). MG had an IC_50_ value of 6.35 µM and exhibited five times greater tyrosinase inhibitory activity compared to kojic acid (IC_50_ = 36.02 µM). AB was found to be inactive. KG displayed significant tyrosinase inhibitory activity with an IC_50_ value of 67.6 µM (Figure 2). KG showed potent activity toward *l*-DOPA with an IC_50_ value of 44.04 µM, which was almost twice that of the positive control (IC_50_ = 79.0 µM). MG showed an acceptable IC_50_ value of 105.57 µM, while AB was inactive. Likewise, 1-methyl-1-cyclohexene moiety itself was inactive regarding both assays (IC_50_ > 1000 µM). However, our result for inhibition of mushroom tyrosinase by KG was significantly different from that of Zheng et al. [12] who demonstrated that KG showed no activity up to 200 µM against monophenolase. Thus, we further studied kinetic and molecular docking analysis to confirm our results.

### 2.2. Enzyme Kinetic Analysis of KG and MG with Monophenolase Activity of Tyrosinase

**Figure 3:**
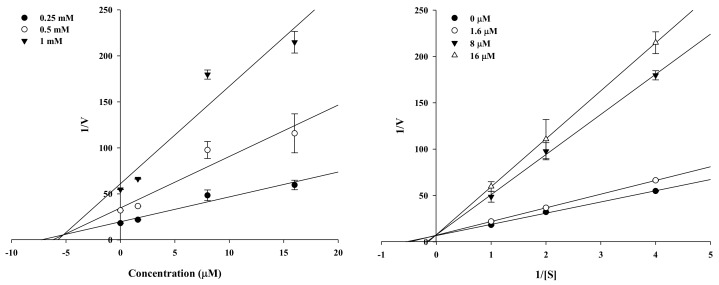
Dixon plots and Lineweaver–Burk plots for mushroom tyrosinase inhibition of mulberrofuran G.

**Figure 4:**
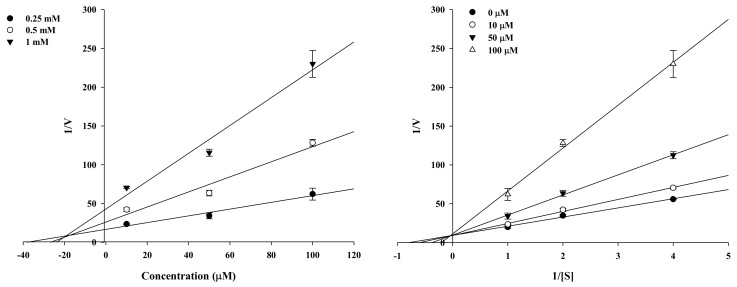
Dixon plots and Lineweaver–Burk plots for mushroom tyrosinase inhibition of kuwanon G.

Kinetic analysis was employed to verify the inhibitory activity of the active compounds and identify the type of inhibition. KG and MG were potent tyrosinase inhibitors as analyzed by Lineweaver–Burk and Dixon plots (Figure 3 and Figure 4). Different concentrations of the substrate (*l*-tyrosine, 1.0, 0.5, and 0.25 mM) along with the various concentrations of KG (10, 50, and 100 µM) and MG (1.6, 8, and 40 µM) were analyzed. The lines of the Dixon plot intersected the y-intercept, which indicated competitive inhibition of tyrosinase with *K_i_* values of 18.66 and 5.19, respectively, for KG and MG (Table 1). The *K_i_* values represent the concentrations required to form an enzyme inhibitor complex, so inhibitors with lower *K_i_* values indicate greater tyrosinase inhibition activity for the development of prophylactic and therapeutic agents.

### 2.3. Molecular Docking Simulation of KG, MG and AB Tyrosinase Inhibition

The enzyme kinetic results indicated that both KG and MG are competitive inhibitors of mushroom tyrosinase. We performed the molecular docking simulation using AutoDock 4.2 to understand the inhibition mechanism of KG and MG. Kojic acid has been used as a selective competitive inhibitor in several studies [31,32,33], but the allosteric inhibition mechanism toward tyrosinase is unclear. Hassani et al. [34] recently reported cinnamic acid as a mixed type inhibitor that interacted with secondary binding sites when the catalytic pocket was occupied with tropolone (co-ligand of 2Y9X). *l*-Tyrosine, kojic acid, and cinnamic acid were used to validate our docking results.

**Figure 5:**
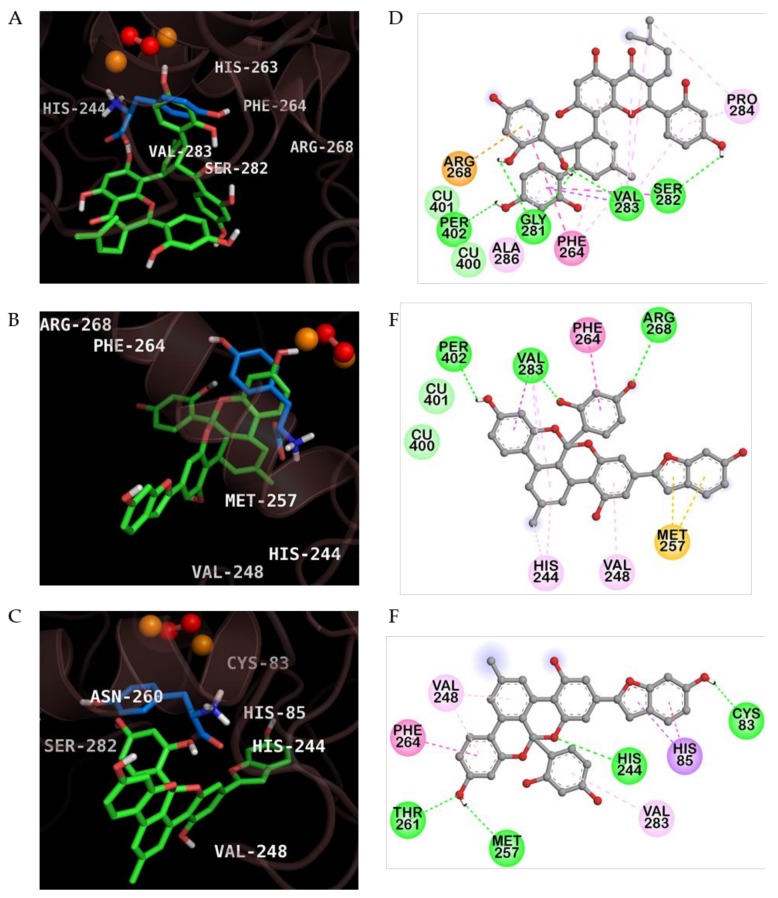
Inhibition mode of kuwanon G (**A**), mulberrofuran G (**B**), and albanol B (**C**) for the oxy-form mushroom tyrosinase catalytic site with *l*-tyrosine (blue stick). Two copper ions and peroxide ions are represented as orange and red spheres, respectively. Tested inhibitors are represented in green stick. 2D diagram of tyrosinase inhibition by kuwanon G (**D**), mulberrofuran G (**E**), and albanol B (**F**).

As shown in Figure 5 and Table 2, MG-enzyme complex showed a low binding energy in the catalytic site of oxy-form mushroom tyrosinase (−7.60 kcal/mol). Two hydrogen bonds were formed between two –OH groups of MG and Arg268 and Val283 residues in the protein-MG complex (Figure 5B,E). In addition, MG interacted with the two peroxide ions via hydrogen bonding interactions (distance: 1.77 and 2.96 Å). In case of protein-KG complex (Figure 5A,D), four hydrogen bonds were observed between –OH groups of KG and Val283, His263, Gly281, and Ser282 residues of enzyme. KG also hydrogen bonded with two peroxide ions (distance: 2.04 and 2.94 Å). Because peroxide ions are important targets for inhibition of oxy-form tyrosinase, MG showed more high binding affinity than KG based on H-bond distance. In addition, two copper ions interacted with MG and KG via van der Waals interactions. We also investigated the interactions between AB and mushroom tyrosinase. Although AB reach to catalytic site of enzyme and possesses the low binding energy (−7.28 kcal/mol), AB could not interacted with copper and peroxide ions. The methyl cyclohexene moiety of KG and MG formed multiple hydrophobic interactions with crucial residues, whereas the methyl benzene ring of AB weakly interacted with the Val248 residue via Pi-alkyl interaction. These in silico computational docking results could explain why the IC_50_ values of MG and KG were lower than that of AB and supported the in vitro experimental kinetic results, which revealed that KG and MG were competitive inhibitors of mushroom tyrosinase.

## 3. Discussion

Tyrosinase is a rate-limiting, copper-containing enzyme that controls the production of melanin in the human body, which can lead to a variety of skin disorders when overproduced [35]. Many natural compounds including quercetin, hydroquinone, chalcones, stilbenes, kojic acid, aloesin, and coumarins have been reported as tyrosinase inhibitors [36]. The present investigation is the first report of MG and AB for enzyme kinetics and molecular docking analysis against mushroom tyrosinase. The inhibitory kinetics and binding mechanisms of KG, MG, and AB from *Morus* species were determined through molecular docking analysis using oxy-form mushroom tyrosinase. Our molecular and structural results clarify the tyrosinase inhibition mechanism of KG and support potential for cosmetic use via tyrosinase inhibition. KG and MG displayed potent inhibitory activity against mono- and diphenolase activity compared to kojic acid. AB did not show any activity, even at a high concentration (350 µM).

KG, MG, and AB have recently attracted extensive research focus. We systematically investigated these three compounds as potential candidates against Alzheimer’s disease [16]. As a part of our ongoing research, we designed *l*-tyrosine and *l*-DOPA oxidation assays to investigate the inhibition activity of these compounds against mushroom tyrosinase. *l*-Tyrosine and *l*-DOPA are consecutive substrates, inducers, and positive regulators of melanogenesis that can regulate melanocyte function through overlapping pathways [37]. Differences among the inhibition activities of these two substrates can be explained by the specific binding properties between the enzyme and substrate. Zheng et al. [12] determined that *l*-tyrosine oxidation was inhibited by MG (IC_50_ = 17.53 µM), which was consistent with our results. In addition to a positive trend, MG was two times more potent than the result obtained in our current study. Slight variation can be attributed to experimental-setup errors. KG showed potent inhibition towards *l*-tyrosine in our study (IC_50_ = 67.6 µM), despite being previously reported as inactive up to 200 µM [12]. Our study refuted this result by providing chemical kinetics and molecular docking data. Chaita et al. [13] also reported that KG showed potent mushroom tyrosinase inhibitory activity (IC_50_ = 27.2 µM) via the *l*-DOPA pathway. These results are in close agreement with our present study (IC_50_ = 44.0 µM).

Molecular mechanistic studies confirmed the tyrosinase inhibition activity of KG and MG via the *l*-tyrosine pathway. The respective double reciprocal Lineweaver–Burk plot revealed the competitive inhibition mechanism of KG and MG, and that both compounds readily bound to the catalytic site. MG had a greater binding affinity than KG as indicated by the lower *K_i_* value of 5.93. For these types of inhibitors, a higher substrate concentration is needed to achieve 50% occupation of the active sites. Kinetic studies revealed that both compounds were competitive inhibitors, indicating that they bind to the enzyme-substrate complex or interact with a specific catalytic site of the enzyme.

Molecular docking studies model the interaction between a small molecule and a protein at an atomic level, which allows characterization of small molecule behavior in the binding site of target proteins and elucidation of fundamental biochemical processes. The binding interactions of MG, KG, and AB from *Morus* species were indicative of tyrosinase inhibition activity. AB had a smaller docking score than kojic acid, but the lack of interactions with copper and peroxide ions and catalytic residues of tyrosinase limited the inhibitory activity. On the other hand, MG and KG interacted with two copper ions and peroxide ions via van der Waals interaction and H-bond, respectively. Multiple hydrophobic interactions were also observed in the protein-KG and –MG complexes. Interestingly, the methyl cyclohexene moiety of KG and MG formed multiple hydrophobic interactions with enzyme residues, whereas the methyl benzene ring of AB weakly hydrophobic interacted with the Val248 residue of enzyme. This is the first report of tyrosinase inhibition activity of MG and KG supported by enzyme kinetic analysis and molecular docking simulation.

Structure–activity relationships might explain the variable activities observed in these flavonoids. We studied the roles of methyl benzene, resorcinol, isoprenyl, and 4-pyrone moieties, focusing on the methyl cyclohexene ring structure. KG is a prenylated flavonoid, while MG and AB are fused benzofurans with specific roles. Zheng et al. [12] demonstrated the importance of isoprenyl moiety, where Kuwanon H contains one additional isoprenyl moiety which attributed to the improved activity as compared to KG. Our study, on the other hand, was not in accordance to this hypothesis, where MG is devoid of isoprenyl moiety, but showed better activity despite the lack of isoprenyl moiety in MG, better monophenolase inhibitory activity than the monoisoprenyl substituted flavone KG.

In another case, previous studies have suggested that the presence of resorcinol moiety in prenylated flavonoids is responsible for the activity. Major compounds kurarinol and kuraridinol from *Sophora flavescens* showed tyrosinase inhibitory activity that was attributed to the resorcinol moiety [38]. The results of our tyrosinase inhibitory assay on the prenylated flavonoid (KG) and fused benzofuran flavonoids (MG and AB) contradicted their hypothesis. While four resorcinol moieties should have increased the activity of the prenylated fused flavonoid KG, the non-prenylated MG with only one resorcinol group was more active. AB on the other hand was also unable to deliver the tyrosinase inhibitory activity, regardless the presence of resorcinol group. Nevertheless, the presence of resorcinol moiety cannot be undermined since the docking study on KG suggested that resorcinol moiety is vital for the activity as it forms a hydrogen bond with GLU 322 which is difficult to break due to hydrophobic interactions. Undoubtedly, resorcinol moiety has been a prime focus since long time, but herein we were compelled to present the additional probability of methyl cyclohexene moiety for the tyrosinase activity. AB comprising methyl benzene moiety in its structure in the catalytic site is unable to bind to the crucial surrounding residues, designating it inactive against tyrosinase inhibition, while the methyl cyclohexene pendant of compounds like KG and MG occupies the catalytic site resulting in the proper orientation with the residues vital for the activity. This fact also explains the reason for the activity of AB, KG, and MG.

Our study suggested that the methyl cyclohexene ring was responsible for inhibitory action against tyrosinase, which is an established hypothesis. The recent evaluation of the essential oils α-pinene and α-terpineol for tyrosinase and melanogenesis inhibition demonstrated positive results [31]. Methyl cyclohexene is a major functional group of α-pinene and α-terpineol and was highly active. This result supported our evidence of the activity of this moiety that is ubiquitous in essential oils. β-caryophyllene from lime mint oil, comprised of the same methyl cyclohexene group, has shown potent skin whitening effects. At 150 µM, β-caryophyllene decreased intracellular tyrosinase activity in B16F10 cells [39]. These results suggest that the methyl cyclohexene group is responsible for tyrosinase inhibitory activity rather than the isoprenyl or resorsinol group itself.

AB has a methyl benzene ring in place of methyl cyclohexene, which explains its inactivity. Benzene has a resonance structure that leads to this inactivity: a large activation energy is required to break the double bonds of unsaturated hydrocarbons. The three double bonds of benzene are delocalized in a pi-system, so a low electron density results in low reactivity. MG forms bonds readily via a methyl cyclohexene functional group instead of a benzene group, which explains the tyrosinase inhibitory activity. The methyl cyclohexene moiety in KG also demonstrated acceptable inhibitory activity. KG has a similar structure to kojic acid, which contains 4-pyrone and might also contribute to its inhibitory activity. Our study supports the functionality of methyl cyclohexene in tyrosinase inhibition.

## 4. Materials and Methods

### 4.1. Chemicals/Reagents and Compounds

KG, MG, and AB were obtained from the root bark of *Morus alba* by Kuk et al. [16] as previously described. *l*-DOPA, kojic acid, and mushroom tyrosinase were purchased from Sigma-Aldrich Co. (St. Louis, MO, USA). *l*-Tyrosine was purchased from Jannssen Chimica (Geel, Belgium). K_2_HPO_4_ was obtained from Junsei Chemical Co. Ltd. (Tokyo, Japan), and KH_2_PO was acquired from Yakuri Pure Chemicals Co. Ltd. (Osaka, Japan). Reagent-grade chemicals and solvents were purchased from commercial sources. Ultra-pure water was used throughout the experiment.

### 4.2. Mushroom Tyrosinase Inhibitory Assay

We investigated the *l*-DOPA and *l*-tyrosine oxidization activity of KG, MG, and AB isolated from root bark of *Morus alba* in our previous study with a modified spectrophotometric method [40,41]. Briefly, 10 µL of a specified concentration of each sample solution (1–500 µg/mL) in 10% dimethyl sulfoxide and 20 µL of mushroom tyrosinase (1000 Units/mL) in a 50 mM phosphate buffer (pH 6.5) were added to 170 µL of an assay mixture in a 96-well microplate. The ratio of 1 mM *l*-tyrosine/*l*-DOPA solution, 50 mM potassium phosphate buffer (pH 6.5), and distilled water was 10:10:9. The reaction mixtures were incubated at 25 °C for 15 s. The absorbance of the mixture at 490 nm was measured using a microplate reader. The degree of inhibition of the sample was expressed as the concentration required for 50% inhibition (IC_50_). One unit (U) of enzymatic activity referred to the amount of an enzyme capable of converting 1 µM of the *l*-tyrosine/*l*-DOPA reaction mixture within 1 min at 280 nm under the specific conditions (25 °C, optimum conditions).

### 4.3. Enzyme Kinetic Analysis with Tyrosinase

We observed the sample activity by decreasing the *l*-tyrosine (1.0, 0.5, and 0.25 mM) substrate concentration to determine the tyrosinase inhibitory mechanism. The reaction mixture consisted of a 20 µL test sample at the following test concentrations: 1.6, 8, and 16 µM for MG; 10, 50, and 100 µM for KG. Tyrosinase inhibition was determined at various concentrations of l-tyrosine substrate (1.0, 0.5, and 0.25 mM) in the absence or presence of the test compound concentrations (1.6, 8, and 16 µM for MG; 10, 50, and 100 µM for KG) using Lineweaver-Burk double reciprocal plots that determine enzyme kinetic parameters including *K_m_* and *V_max_*. The enzymatic procedures consisted of the previously described tyrosinase assay methods. The inhibition constant (*K_i_*) was determined from the interpretation of Dixon plots.

### 4.4. Tyrosinase Molecular Docking Simulations

The H-subunit (residues 2–392) of deoxy-form mushroom tyrosinase protein was obtained from the RCSB Protein Data Bank (ID: 2Y9X) [42]. Small molecules, including water, holmium (Ho) atoms, and tropolone, excluding copper (II) ions, were removed from the target enzyme. Binuclear copper-binding catalytic site of H-subunit of 2Y9X was slightly modified to fulfill the oxy-form enzyme [43]. All hydrogens were added. AutoDock 4.2 program was used to predict the ligand-protein interactions [44,45]. The copper ion parameters were prepared and added to run AutoGrid 4. The 3D structures of KG, MG, AB, *l*-tyrosine, kojic acid, and cinnamic acid were downloaded from PubChem Compound (NCBI), with compound CIDs of 5281667, 196583, 480819, 6057, 3840, and 444539, respectively. Kojic acid and cinnamic acid [34] were used as the reported catalytic and mixed type inhibitors against mushroom tyrosinase, respectively, and their binding sites were used to validate the results of AutoDock 4.2 docking analysis. The protein-ligand interactions were visualized and analyzed using PyMOL 1.7.4 (Schrodinger, LLC, Cambridge, MA, USA) for 3D models, and Discovery Studio Visualizer 16.1 (Accelrys, San Diego, CA, USA) was used for 2D diagrams.

### 4.5. Statistical Analysis

Differences between the control group and the test group were determined using Student’s *t*-test (Sysat Inc., Evanston, IL, USA), and *P* < 0.05 was used as a statistically significant cut-off value. All results were expressed as the mean ± S.E.M. of triplicate experiments.

## 5. Conclusions

To sum up, KG and MG have attracted much interest in recent years, which inspired us to explore their potential cosmetic applications through computational studies and structural elucidation. In addition, we have examined the inhibitory activities of KG, MG, and AB against both pathways of melanogenesis (monophenolase and diphenolase activities). Enzyme kinetic and computational assessment suggested that MG and KG were potent tyrosinase competitive inhibitors via H-bonding interaction with peroxide ion and van der Waals interaction with copper ions at the active site. This is the first report of MG, KG, and AB for enzyme kinetic mechanisms and molecular docking analysis to the best of our knowledge. The controversial inhibitory activity of KG has been resolved via kinetics and docking simulation studies, and the importance of the methyl cyclohexene functional group has been explored. Further studies of this moiety are necessary. Our results indicate that these active flavonoids might be promising candidates as cosmetic agents. However, further studies are needed to fully characterize the underlying mechanism responsible for the effects of KG and MG on murine or mammalian cell based assays.

## Figures and Tables

**Table 1:** Inhibitory activities of the compounds isolated from the root bark of *Morus alba* on mushroom tyrosinase.

Compounds	IC_50_ (μM) Value ^a^		Inhibition Mode ^b^	Inhibition Constant (*K_i_*, μM) ^c^
	*l*-Tyrosine	*l*-DOPA		
Kuwanon G	67.6 ± 2.11	44.0 ± 3.73	Competitive	18.7
Mulberrofuran G	6.35 ± 0.45	105.6 ± 1.85	Competitive	5.19
1-Methyl-1-cyclohexene	>1000	>1000	NT ^e^	NT
Albanol B	>350	>350	NT	NT
Kojic acid ^d^	36.0 ± 0.88	79.0 ± 0.06	NT	NT

^a^ The 50% inhibition concentration (μM) is calculated from a log-dose inhibition curve and expressed as the mean ± SEM of triplicate experiments. ^b^ Determined by Lineweaver–Burk plot. ^c^ Determined by Dixon plot. ^d^ Positive control. ^e^ Not tested. ***l***-DOPA: l-3,4-dihydroxyphenylalanine.

**Table 2:** Molecular interaction of oxy-form mushroom tyrosinase active site with three compounds as well as positive controls.

Compounds	Binding Energy (Kcal/mol)	H-Bond Interaction	Van der Waals Interaction	Hydrophobic Interaction	Others
Kuwanon G	−6.95	Per402, Val283, His263, Gly281, Ser282	Cu400, Cu401	Pi-sigma: Val283 Pi-Pi Stacked: His263 Pi-Pi T-Shaped: Phe264 Amide-Pi Stacked: Val283, Ser282 Alkyl: Val283, Pro284 Pi-Alkyl: Phe264, Val283, Pro284, Ala286	Pi-Cation: Arg268
Mulberrofuran G	−7.60	Per402, Arg268, Val283	Cu400, Cu401	Pi-sigma: Val283 Pi-Pi T-Shaped: Phe264 Alkyl: Val283 Pi-Alkyl: His244, Val248	Pi-Sulfur: Met257
Albanol B	−7.28	Cys83, His244, Met257, Thr261	‒	Pi-Sigma: His85 Pi-Pi T-shaped: His85, Phe264 Pi-Alkyl: Val248, Val283	‒
*l*-Tyrosine ^a^	−6.31	His244, Asn260, Met280, Glu256 (Salt-bridge)	Per402, Cu400, Cu401	Pi-Sigma: Val283 Pi-Pi Stacked: His263 Pi-Alkyl: Ala286	‒
Kojic acid ^b^	−5.5	Met280	Cu400, Cu401	Pi-Sigma: Val283 Pi-Pi T-Shaped: His263 Pi-Alkyl: Ala286	
Cinnamic acid ^b,c^	−6.20	Gln307, Asp312, Glu356	Tyr314	Pi-Pi Stacked: Trp358	‒

^a^ Reported competitive ligand. ^b^ Reported competitive type inhibitor.^b^ Reported mixed type inhibitor. ^c^ Interaction of cinnamic acid to allosteric site, while troplone (co-ligand of 2Y9X) is sited in catalytic pocket.
